# The effect of duration of exercise at the ventilation threshold on subjective appetite and short-term food intake in 9 to 14 year old boys and girls

**DOI:** 10.1186/1479-5868-6-66

**Published:** 2009-10-09

**Authors:** Natalie C Bozinovski, Nick Bellissimo, Scott G Thomas, Paul B Pencharz, Robert C Goode, G Harvey Anderson

**Affiliations:** 1 Department of Nutritional Sciences, Faculty of Medicine, University of Toronto, FitzGerald Building, 150 College St, Toronto, ON M5S 3E2, Canada; 2 Graduate Department of Exercise Sciences, Faculty of Physical Education and Health, University of Toronto, 55 Harbord St, Toronto, ON M5S 2W6, Canada; 3 Department of Paediatrics, Faculty of Medicine, University of Toronto, Toronto, Canada; 4 Department of Applied Human Nutrition, Faculty of Professional Studies, Mount Saint Vincent University, 166 Bedford Highway, Halifax, NS B3M 2J6, Canada

## Abstract

**Background:**

The effect of exercise on subjective appetite and short-term food intake has received little investigation in children. Despite a lack of reported evaluation of short-duration activity programs, they are currently being implemented in schools as a means to benefit energy balance. The purpose of this study was to determine the effect of duration of exercise at the ventilation threshold (VeT) on subjective appetite and short-term food intake in normal weight boys and girls aged 9 to 14 years.

**Methods:**

On 4 separate mornings and in random order, boys (n = 14) and girls (n = 15) completed 2 rest or 2 exercise treatments for 15 (short-duration; SD) or 45 min (long-duration; LD) at their previously measured VeT, 2 h after a standardized breakfast. Subjective appetite was measured at regular intervals during the study sessions and food intake from a pizza meal was measured 30 min after rest or exercise.

**Results:**

An increase in average appetite, desire to eat, and hunger (p < 0.05) was attenuated by SD exercise, but was further increased (p < 0.05) by LD exercise. However, food intake after SD and LD exercise was similar to after rest in both boys and girls (p = 0.55). The energy cost of SD and LD exercise resulted in a lower net energy balance compared to resting during the study measurement period in boys (SD: Δ = -418 ± 301 kJ; LD: Δ = -928 ± 196 kJ) and in girls (SD: Δ = -297 ± 105 kJ; LD: Δ = -432 ± 115 kJ).

**Conclusion:**

Neither SD nor LD exercise at the VeT increased short-term food intake and SD exercise attenuated increases in appetite. Thus, SD exercise programs in schools may be an effective strategy for maintaining healthier body weights in children.

## Background

Current policies and programs geared towards children are promoting short-duration bouts of exercise during the school day to improve fitness and body weight. Since 2005 in Ontario, all elementary schools are required to include at least 20 min of daily physical activity during instructional time for children aged 6 - 14 years [1]. In the United States, a program called Take 10! instructs teachers on including 10 min of age-appropriate aerobic activities into the school day [2]. These short-duration programs of activity are attractive because they are applicable to children of all ages, require little training, and can be done in the classroom. Take 10! has reached such popularity that an adaptation of this program called the Happy 10! was recently implemented in schools in Beijing [3].

However, evaluation of the effect of short-duration activities on appetite, energy intake, or energy balance in children is limited to three reports. In one study, 2 bouts of exercise at either low- (50% peakVO_2 _for ~60 min) or high-intensity (75% peakVO_2 _for ~40 min) did not affect total energy intake over the day in 9 - 10 year old normal weight girls [4]. Lunch energy intake was lower after one bout of the low- but not high-intensity exercise, compared with the no exercise control. Prospective consumption was lower mid-afternoon for the high- when compared to the low-intensity exercise condition and control, but was otherwise unaffected by exercise. Similarly, when two bouts daily of high-intensity exercise (75% peak VO2 for ~40 min) repeated for 2 days was imposed in normal weight and overweight 11 year old girls, energy intake was not affected during exercise days or the two days following [5]. However, the overweight, but not normal weight girls, rated their appetite as being higher after exercise when compared to before exercise. Increased appetite was also found following 12 minutes of a submaximal incremental exercise test assessing the ventilation threshold (VeT), in 9 to 14 year old normal weight boys [6]. In the latter study food intake was not measured, so it remains uncertain whether food intake would have increased. Nevertheless these studies raise uncertainty of the benefit to energy balance of short-duration exercise, as could occur in the classroom. An increase in appetite followed by increased food intake would easily counter any benefit to energy balance.

Therefore, the purpose of this study was to examine the effect of duration of an acute bout of exercise at the VeT on subjective appetite and short-term food intake in normal weight boys and girls aged 9 to 14 years. We hypothesized that a single bout of SD or LD exercise at the VeT will not increase short-term food intake in children.

## Methods

### Subjects

Fourteen normal weight boys and fifteen normal weight girls (between the 15^th ^and 85^th ^BMI percentile [7]) between the ages of 9 and 14 years and with no differences in baseline characteristics (Table 1) were recruited from the Toronto District School Board (TDSB), Toronto Catholic District School Board (TCDSB), and the University of Toronto Schools (UTS) via a recruitment letter sent home to parents. The Human Subjects Review Committee, Ethics Review Office, University of Toronto Canada, the TDSB, and TCDSB approved this study.

**Table 1:** Baseline characteristics of test subjects

**Subject Characteristics**			
	**Boys**	**Girls**	P-value*
**N**	14	15	
**Age (years)**	12.6 ± 0.3	11.7 ± 0.4	0.075
**Height (cm)**	156.0 ± 3.7	150.9 ± 2.6	0.28
**Weight (kg)**	46.5 ± 2.8	41.9 ± 2.5	0.24
**BMI (%ile)**	55.9 ± 4.3	52.8 ± 6.2	0.69
Fat mass** (kg)	10.5 ± 0.8	10.1 ± 1.3	0.80
Fat mass** (%)	22.7 ± 1.3	22.8 ± 2.0	0.98
**Fat free mass (kg)**	36.0 ± 2.4	31.8 ± 1.4	0.15
**Fat free mass (%)**	77.3 ± 1.3	77.2 ± 2.0	0.98
**Ventilation Threshold (mL·min^-1^)**	1067.5 ± 90.7	933.1 ± 42.1	0.18
**Ventilation Threshold** **(mL·kg^-1^·min^-1^)**	23.0 ± 1.2	22.2 ± 0.9	0.59

Data are means ± SEM (*unpaired t-test).

**Fat mass determined from the sum of skinfold measurements at four points [8].

Boys and girls born at full-term and normal weight and not dieting, taking medication, or having significant learning, behavioural, or emotional difficulties were selected for inclusion in the study. An appointment was then made for a screening session with the child and parent at the Department of Nutritional Sciences where informed written consent was obtained from the parent and written assent from the child. Height (m), weight (kg), triceps, biceps, supra-ilial, and subscapular skinfold thickness (mm), were obtained using a Harpenden skinfold calliper and recorded to the nearest 0.1 mm. The sum of the 4 skinfold measurements was used to estimate percent fat mass from a sex specific regression equation [8], according to the procedures previously described [6,9-11]. The parent and child were given a tour of the facility to familiarize them with the tasks and expectations of the study.

### Study design and procedures

A within-subject repeated measures design was used. Boys and girls completed, in random order, on 4 separate weekend mornings 2 rest or 2 exercise treatments for 15 min (short-duration; SD) or 45 min (long-duration; LD), 2 hours after the consumption of a standardized breakfast of milk, cereal, and orange juice. Visual analogue scales (VAS) to assess motivation to eat and physical comfort were completed at baseline (0 min), and at regular intervals up to lunch, and immediately after. A pizza lunch was provided to subjects 30 min after completion of each of the treatment conditions. Following lunch, children completed a VAS assessing the palatability of the pizza meal.

### Measurement of Food Intake

As previously described in greater detail elsewhere [6,9-11], boys and girls arrived at the Department of Nutritional Sciences at 10:00 am, 11:00 am, or 12:00 pm, and consistent between trials for each participant. Participants arrived 2 hours after consuming the standardized breakfast at home. The standardized breakfast consisted of Parmalat^® ^fat-free skim milk (250 mL, 376 kJ), Honey Nut Cheerios^® ^(26 g, 418 kJ donated by General Mills, Inc.) and Tropicana Orange Juice^® ^(236 mL, 460 kJ). However, one girl was served original Cheerios^® ^(28 g, 418 kJ) because she found the honey nut variety to be unpalatable. Participants and their parents were asked to verify consumption of the breakfast prior to beginning the study session and to ensure no other food was consumed. If there was any deviation from the protocol, participants were sent home and re-scheduled to return the following week.

Upon arrival and at 15 min intervals up to lunch, participants completed VAS questionnaires (between 0 and 100 mm) measuring their motivation-to-eat and physical comfort. The motivation-to-eat VAS included 4 questions (i) how strong is your desire to eat? ("very weak" to "very strong"), (ii) how hungry do you feel? ("not hungry at all" to "as hungry as I've ever felt"), (iii) how full do you feel? ("not full at all" to "very full"), and (iv) how much food do you think you can eat? (prospective food consumption, PFC; "nothing at all" to "a large amount") and the physical comfort VAS asked how well do you feel? ("not well at all" to very well") as used previously [6,9-11].

Thirty minutes after the completion of the rest or exercise protocols children were individually seated in their own cubicle in a room free of most external cues. Participants were then served an ad libitum pizza lunch of the variety selected by them at the screening session along with a 500 mL bottle of spring water (Danone Crystal Springs). Children were informed that additional hot trays would be presented at regular intervals and were instructed to eat until they were "comfortably full." Upon completion of the lunch, participants completed the post-meal motivation-to-eat, physical comfort, and palatability of the pizza meal VAS questionnaires.

Two varieties of Deep 'n' Delicious 5" diameter pizza (averaging 920 kJ) were used; pepperoni and three-cheese pizzas donated by McCain^® ^Foods (McCain^® ^Canada Ltd., Florenceville, ON). Pepperoni pizza slices (102 g) contained 11 g of protein, 6 g of fat, and 27 g of carbohydrate (210 kcal). Each three-cheese pizza slice (96 g) contained 12 g of protein, 7 g of fat, and 30 g of carbohydrate (230 kcal). The amount left after the meal was subtracted from the initial weight to determine the net weight consumed (in grams). The energy consumed (kJ) was calculated by weighing each variety of pizza separately and converting the net weight consumed in grams to kJ consumed by use of information provided by the manufacturer (McCain^® ^Foods Ltd.). The 500 mL bottle of spring water (Danone Crystal Springs) served with the meal was also weighed before and after to determine intake. Additional bottles of water were provided to participants when requested.

### Eating Behaviour Assessment

The Dutch Eating Behaviour Questionnaire was administered to all participants to determine restrained eating and disinhibition [12]. Younger participants who may have had difficulty interpreting the language of the questionnaire had assistance completing the survey by either a parent or research assistant who interpreted the questions for him or her.

### Determination of VeT

VeT was determined using a graded submaximal fitness test walking protocol on a weeknight, separate from food intake sessions and at least 3 hours after their last meal. The VeT is defined as the point during graded exercise where ventilation increases disproportionately to increases in oxygen consumption [13]. The VeT of each child was used as the target exercise intensity and chosen as a marker of cardiovascular fitness because it is a sensitive indicator of physical fitness at a heart rate of 170 bpm and does not require a maximal effort during testing procedures. In addition, previous reports indicate that a plateau in VO_2max _during incremental exercise will only occur in approximately 21 to 60 percent of children [14], which indicates that maximal fitness testing may be suboptimal for this age group. Furthermore, the VeT has been referred to as a valid and useful measure of aerobic fitness in children [15].

Respiratory gas exchange was measured during a continuous, progressive increase in ramp speed and grade on a motorized treadmill (Trackmaster TMX 425 CP, Newton, USA) until approximately 80 - 85% maximum heart rate (HR) was achieved. One of three walking protocols was selected for each participant based on their reported level of physical activity, height, weight, and age in order to ensure an appropriate work rate for measurement of the VeT. Ratings of perceived exertion (RPE) were obtained using the Borg Scale [16] at 1 min intervals during the fitness test, following a three minute warm-up. The VeT was determined as previously described [6,11].

### Exercise Protocol

Participants walked on a LifeFitness T5.5 Treadmill at an individually determined speed and incline designed to employ the metabolic demand of exercise at the VeT. Before commencement of exercise, subjects were fitted with a LifeFitness HR monitor (POLAR^® ^T34, Kempele, Finland) by chest-strap to provide an indication of exercise intensity. Children were supervised by a research volunteer throughout the duration of exercise who also recorded HR and RPE as determined by 15 point Borg Scale [16] at the end of each minute of exercise to confirm children were exercising at their individual VeT.

### Rest Protocol

During resting control treatments and the 30 min period following rest and exercise treatments, children engaged in age-appropriate board and card games. Research volunteers supervised the children at all times. Study personnel were instructed to distract the children from engaging in food related discussions. For instance, if a child asked when they would be eating a research volunteer would suggest playing a game together, or start a conversation with the child about something unrelated to food.

### Calculations and Statistical analyses

Energy expenditure during the exercise treatment was calculated as energy expended above resting metabolic rate using the American College of Sports Medicine (ASCM) walking equation [17].



The resting metabolic rate of 3.5 mL·kg^-1^·min^-1 ^was first removed from the calculation. VO_2 _(mL·kg^-1^·min^-1^) was calculated using the prescribed speed and grade during treadmill exercise. It was then multiplied by body weight (kg) to obtain VO_2 _(mL·min^-1^) which was divided by 1000 to obtain VO_2 _(L·min^-1^) and then multiplied by 5 to determine energy expenditure in kcal·min^-1^. Finally this number was multiplied by the duration of exercise in minutes to obtain an estimate of exercise induced energy expenditure.

An average appetite score was calculated at each time of measurement for each treatment by the formula:



which reflects the 4 questions on the motivation-to-eat VAS as used previously [6,9-11].

Food intake minus exercise energy expenditure was calculated using the following formula:



Net energy balance (NEB) for the duration of the study measurement period was calculated as follows:



Baseline subject characteristics were analyzed between groups by unpaired t-test. Food and water intake, palatability, and food intake minus exercise energy expenditure were analyzed using a within-subject repeated measures 3 × 2 factorial design using the PROC MIXED procedure with treatment (rest vs. exercise), duration (SD vs. LD), and sex (boys vs. girls) as main factors.

VAS for average appetite and individual appetite scores were analyzed as the change from baseline using a within-subject repeated measures three way design using the PROC MIXED procedure with time (SD: 0, 15, 30, 45, 75/85 min; LD: 0, 15, 30, 45, 60, 75, 105/115 min), treatment, and sex as main factors. VAS data was analyzed separately for SD and LD because the study length differed.

Because energy expenditure, HR, and RPE were only measured during the exercise sessions, they were each analyzed using a within-subject repeated measures two way design using the PROC MIXED procedure with treatment and sex as main factors. NEB was also analyzed using a two way design with duration and sex as main factors.

Statistical significance was defined as p < 0.05. Correlations on dependent measures were conducted using Pearson's correlation coefficients. SAS version 9.13 (Statistical Analysis Systems, SAS Institute Inc., Carey, NC) was used to perform all statistical analyses.

## Results

### Subjective appetite during short-duration sessions

**Figure 1:**
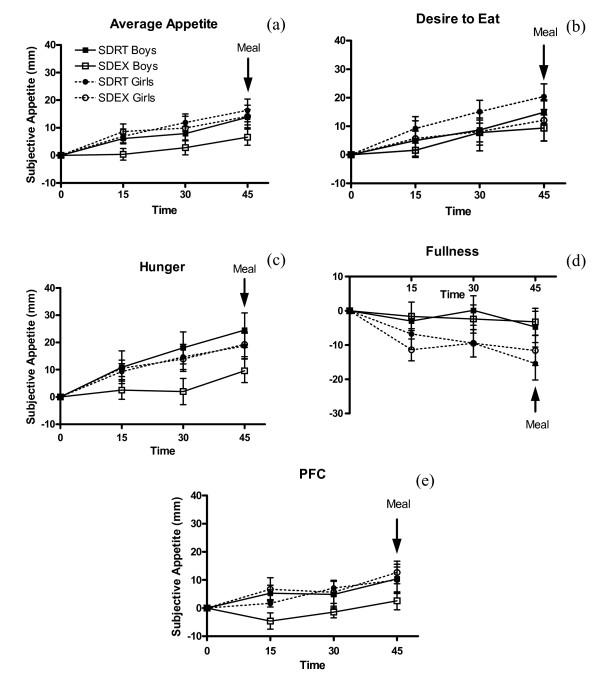
**Effect of short-duration exercise at the VeT on subjective appetite scores**. ^1^Average appetite (a) desire to eat (b) hunger (c) fullness (d) PFC (e) at 15, 30, and 45 min during short-duration sessions. ^2^SDRT = short-duration rest, SDEX = short-duration exercise, PFC = prospective food consumption. ^3^Change from baseline appetite scores increased with time for average appetite (p = 0.0005), desire to eat (p = 0.011), hunger (p = 0.0027), and PFC (p = 0.0047), and subjective fullness decreased (p < 0.0001). SDEX attenuated the increase in average appetite (p = 0.027), desire to eat (p = 0.049), and hunger (p = 0.0072) when compared with SDRT, but did not affect fullness (p = 0.98) or PFC (p = 0.15). Sex was not a factor on change from baseline average appetite (p = 0.11), desire to eat (p = 0.41), hunger (p = 0.46), fullness (p = 0.81), or PFC (p = 0.22). However, there was a significant treatment × sex interaction on PFC (p = 0.013). ^4^Test meal began at 45 min.

Change from baseline scores increased with time for average appetite (p = 0.0005), desire to eat (p = 0.011), hunger (p = 0.0027), and PFC (p = 0.0047), and subjective fullness decreased with time (p < 0.0001) (Figure 1). SDEX attenuated the increase in average appetite (p = 0.027), desire to eat (p = 0.049), and hunger (p = 0.0072) when compared with SDRT but did not affect fullness (p = 0.98) or PFC (p = 0.15). Sex was not a factor on change from baseline average appetite (p = 0.11), desire to eat (p = 0.41), hunger (p = 0.46), fullness (p = 0.81) or PFC (p = 0.22). There was a significant treatment × sex interaction on subjective hunger (p = 0.0052), driven by the lack of increase in hunger from baseline to 30 min in boys during SDEX. There was also a significant treatment × sex interaction on PFC (p = 0.013) because PFC decreased from baseline to 15 min in boys during SDEX.

### Subjective appetite during long-duration sessions

**Figure 2:**
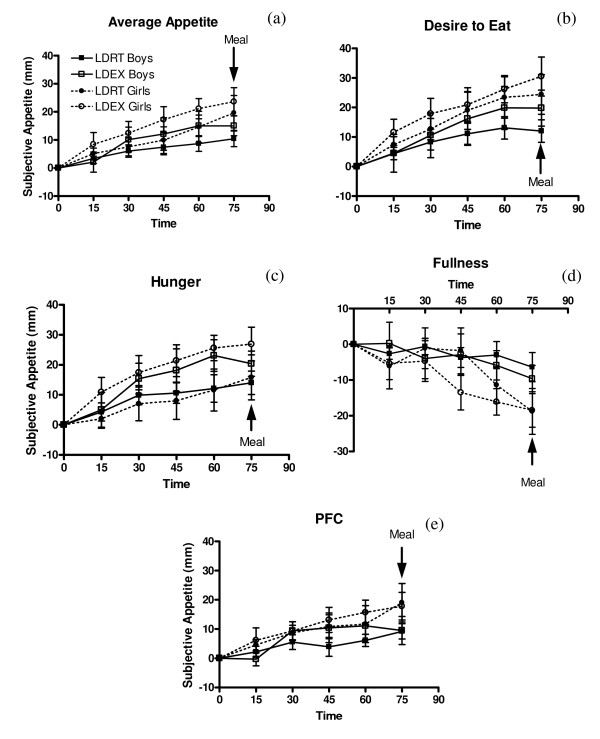
**Effect of long-duration exercise at the VeT on subjective appetite scores**. ^1^Average appetite (a) desire-to-eat (b) hunger (c) fullness (d) PFC (e) at 15, 30, 45, 60, and 75 min during long-duration sessions. ^2^LDRT = long-duration rest, LDEX = long-duration exercise, PFC = prospective food consumption. ^3 ^Change from baseline average appetite (p < 0.0001), desire to eat (p < 0.0001), hunger (p < 0.0001), and PFC (p < 0.0001) increased with time, and fullness decreased (p < 0.001). LDEX stimulated a greater rate of increase in average appetite (p = 0.0045), desire to eat (p = 0.047), and hunger (p < 0.0001) compared with LDRT, however there was no effect on fullness (p = 0.19) or PFC (p = 0.18). Sex was not a factor on change from baseline average appetite (p = 0.22), desire to eat (p = 0.15), hunger (p = 0.80), fullness (p = 0.17), or PFC (p = 0.17). ^4^Test meal began at 75 min.

Change from baseline average appetite (p < 0.0001), desire to eat (p < 0.0001), hunger (p < 0.0001), and PFC (p < 0.0001) all increased with time, and fullness decreased (p < 0.001) (Figure 2). LDEX stimulated a greater rate of increase in average appetite (p = 0.0045), desire to eat (p = 0.047), and hunger (p < 0.0001) compared with LDRT, however there was no effect on fullness (p = 0.19) or PFC (p = 0.18). Average appetite (p = 0.22), desire to eat (p = 0.15), hunger (p = 0.80), fullness (p = 0.17), and PFC (p = 0.17) were unaffected by sex, and there were no significant interactions among time, treatment, and sex.

### Physical comfort

**Figure 3:**
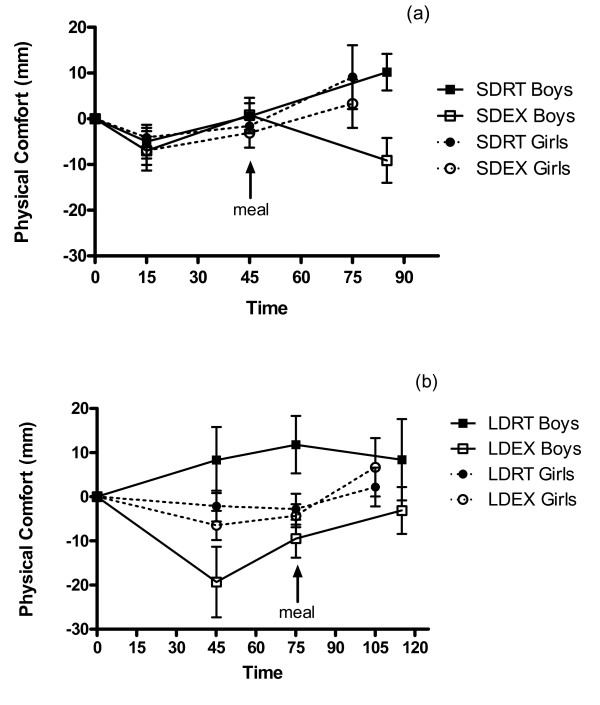
**Physical comfort during (a) short- and (b) long-duration sessions in boys and girls**. ^1^SDRT = short-duration rest, SDEX = short-duration exercise, LDRT = long-duration rest, LDEX = long-duration exercise. ^2 ^Change from baseline physical comfort increased over time during SD (a) (p = 0.036), but not LD (b) (p = 0.26) sessions. Neither treatment (p = 0.49), nor sex (p = 0.58) was a factor affecting physical comfort during SD sessions. Physical comfort during LD sessions was affected by treatment (p < 0.0001), but not sex (p = 0.73), and there was a significant treatment × sex interaction (p = 0.0007). ^3^Test meals began at 45 min (a) and 75 min (b).

Change from baseline physical comfort increased over time during SD (p = 0.036), but not LD sessions (p = 0.26) (Figure 3). Neither treatment (p = 0.49), nor sex (p = 0.58) was a factor affecting physical comfort during SD sessions. However, physical comfort during LD sessions was affected by treatment (p < 0.0001), with exercise decreasing ratings of physical comfort. There was no main effect of sex (p = 0.73), but there was a significant treatment × sex interaction (p = 0.0007) because the boys, compared with girls, reported lower physical comfort after the LDEX.

### Food intake, water intake, and palatability

Food intake at the test meal was not affected by treatment (p = 0.94) or duration (p = 0.55), and there were no significant interactions among treatment, duration, or sex. Sex was a factor on food intake at the test meal (p = 0.0029) with girls, compared with boys, consuming less energy (Table 2).

**Table 2:** Effect of exercise on food and water intake, palatability, EE, and NEB in boys and girls^1^

**Sex**	**Boys**				**Girls**			
**Treatment**	**SDRT**	**SDEX**	**LDRT**	**LDEX**	**SDRT**	**SDEX**	**LDRT**	**LDEX**

Food intake^2 ^(kJ)	5476 ± 527	5342 ± 389	5467 ± 468	5392 ± 443	3645 ± 351	3591 ± 301	3579 ± 350	3876 ± 334

Water intake^3 ^(g)	253.4 ± 45.8	261.1 ± 46.2	259.8 ± 51.5	354.6 ± 36.6	163.7 ± 44.0	164.1 ± 47.9	137.6 ± 43.0	195.8 ± 43.5

Palatability^4 ^(mm)	76.5 ± 7.3	75.1 ± 7.5	74.6 ± 7.3	75.4 ± 7.1	76.1 ± 5.2	75.8 ± 5.2	75.3 ± 4.6	75.9 ± 5.4

Exercise energy expenditure^5 ^(kJ)	0	284 ± 25	0	853 ± 79	0	242 ± 13	0	730 ± 37

Food intake minus exercise energy expenditure^6 ^(kJ)	5476 ± 527	5058 ± 376	5467 ± 468	4539 ± 405	3645 ± 351	3348 ± 301	3578 ± 351	3146 ± 337

Net energy balance^7 ^(kJ)	-418 ± 301		-928 ± 196		-297 ± 105		-432 ± 115	

^1^Data are means ± SEM.

^2 ^Food consumed at a pizza meal 30 minutes after end of treatment (rest or exercise). Food intake was not affected by treatment (p = 0.94), or duration (p = 0.55), but there was an effect of sex (p = 0.0029).

^3^Water consumed at a pizza meal 30 minutes after end of treatment (rest or exercise). Water intake was affected by exercise (p = 0.013), but not duration (p = 0.091), or sex (p = 0.054) and there was a treatment × duration interaction (p = 0.023).

^4^Palatability of the pizza meal. Palatability of the test meal was not affected by treatment (p = 0.95), duration (p = 0.74), or sex (p = 0.96).

^5^Energy expenditure was affected by duration (p < 0.0001), but not sex (p = 0.11).

^6^Food intake minus exercise energy expenditure was affected by treatment (p < 0.0001) and sex (p = 0.003), but not duration (p = 0.071).

^7 ^Net energy balance = [{Food intake after exercise (kJ) - Exercise energy expenditure (kJ)} - (Food intake after rest treatment (kJ)]. Net energy balance was not affected by duration (p = 0.13), or sex (p = 0.093), and there was no duration × sex interaction (p = 0.36).

Water intake at the test meal was affected by treatment (p = 0.013), but not by duration (p = 0.091), or sex (p = 0.054) (Table 2). There was also a significant treatment × duration interaction (p = 0.023) because water intake was higher after LDEX compared with the other sessions.

Palatability of the test meal was not affected by treatment (p = 0.95), duration (p = 0.74), or sex (p = 0.96), and there were no significant interactions (Table 2).

### HR, RPE, and energy expenditure

HR and RPE were analyzed by taking the mean values during the last 10 min of exercise for each subject at each exercise session.

HR was affected by duration (p = 0.027) of exercise, but not sex (p = 0.51). There was also a significant duration × sex interaction (p = 0.043), driven by the increased HR in boys during LDEX when compared to SDEX, while HR in girls was unaffected by duration (Table 3).

**Table 3:** Effect of short- and long-duration exercise on HR and RPE in boys and girls^1^

**Sex**	**Boys**		**Girls**	
**Treatment**	**SDEX**	**LDEX**	**SDEX**	**LDEX**

HR (bpm)^1^	145 ± 4	155 ± 4	153 ± 5	154 ± 4
RPE (Borg Scale units)^2^	11 ± 1	13 ± 1	12 ± 1	15 ± 1

Data are means ± SEM

^1^HR was affected by duration (p = 0.027) of exercise, but not sex (p = 0.51) and there was a significant duration x sex interaction (p = 0.043).

^2 ^RPE was affected by duration (p = 0.0004) of exercise, but not sex (p = 0.08), and there was no significant duration x sex interaction (p = 0.6).

RPE was affected by duration (p = 0.0004) but not sex (p = 0.08), and with no significant duration × sex interaction (p = 0.6) (Table 3).

Energy expenditure was higher after LDEX compared with SDEX (p < 0.0001), but did not differ between boys and girls (p = 0.11), and there was no duration × sex interaction (Table 2).

### Food intake minus exercise energy expenditure and NEB

Food intake minus exercise energy expenditure was affected by treatment (p < 0.0001) and sex (p = 0.003), but not by duration (p = 0.071), and there were no significant interactions among treatment, duration or sex (Table 2).

NEB was not affected by duration (p = 0.13) or sex (p = 0.093) and there was no duration × sex interaction (p = 0.36) (Table 2).

### Associations among body composition, VeT, disinhibition, and food intake

In boys, body weight (kg) was positively associated with fat mass (%) (r = 0.97, p < 0.0001), but not fat free mass (kg) (r = -0.061, p = 0.84) and positively associated with VeT per kg body weight (r = 0.64, p = 0.015). Fat mass (%) was inversely associated with VeT per kg of body weight (r = 0.73, p = 0.0032).

In girls, body weight (kg) was positively associated with fat mass (%) (r = 0.93, p < 0.0001), fat free mass (kg) (r = 0.94, p < 0.0001), and inversely associated with VeT per kg body weight (r = -0.67, p = 0.0064). Fat mass (%) was inversely associated with VeT per kg of body weight (r = -0.66, p = 0.077).

Body weight (kg) (r = 0.58, p = 0.029; r = 0.71, p = 0.0044), fat free mass (kg) (r = 0.57, p = 0.032; r = 0.73, p = 0.0029), and VeT per kg body weight (r = 0.58, p = 0.030; r = 0.72, p = 0.0037) were positively associated with food intake (kJ) and disinhibition, respectively in boys, but not in girls. Disinhibition was positively correlated with food intake (kJ) in boys (r = 0.54, p = 0.047) but not girls (r = -0.039, p = 0.89).

### Association between subjective average appetite and food intake

In girls, but not boys, food intake (kJ) was positively correlated with subjective appetite at each time point during SD sessions (Table 4). During LD sessions, subjective appetite was correlated with food intake at all time points in girls, but was only correlated with food intake at the fifteen minute measurement time point in boys (Table 5).

**Table 4:** Association between subjective average appetite and food intake in boys and girls during short-duration sessions^1^

	**Boys**		**Girls**	
**Time**	**r**	**P**	**r**	**p**

**0**	0.029	0.88	0.48	0.0076
**15**	-0.027	0.89	0.49	0.0056
**30**	-0.0044	0.98	0.62	0.0002
**45**	-0.00087	0.99	0.70	< 0.0001

^1^Pearson correlation coefficients, n = 28 boys; n = 30 girls.

**Table 5:** Association between subjective average appetite and food intake in boys and girls during long-duration sessions^1^

	**Boys**		**Girls**	
**Time**	**r**	**P**	**R**	**p**

**0**	0.36	0.063	0.43	0.017
**15**	0.40	0.033	0.44	0.014
**30**	0.28	0.15	0.38	0.040
**45**	0.16	0.41	0.49	0.0066
**60**	0.14	0.47	0.56	0.0013
**75**	0.25	0.21	0.64	0.0002

^1^Pearson correlation coefficients, n = 28 boys; n = 30 girls.

## Discussion

The results from this study supported the hypothesis that a single bout of SDEX or LDEX at the VeT does not increase short-term food intake in children. Thus the application of SDEX during the school day may contribute to healthier body weights in children.

In the current study, walking on a treadmill at a moderate intensity for 15 min and 45 min duration at an estimated energy expenditure of approximately 260 kJ and 790 kJ respectively did not significantly affect food intake at a test meal thirty minutes after exercise when compared with the resting control, consistent with the literature in both children [4,5] and adults [18-21]. As a result, the energy expended during exercise resulted in a lower net energy balance for the duration of the experimental period. Fifteen and forty-five minutes of exercise reduced NEB in boys by approximately -418 kJ and -928 kJ, respectively and in girls by approximately -297 kJ and -432 kJ, respectively.

There is some indication, however, that girls compared with boys, have a lower or diminished ability to tolerate energy deficits caused by LDEX. Subjective appetite was similarly increased by LDEX in boys and girls, but there was a trend in girls only to increase food intake after LDEX by approximately 297 kJ. Unfortunately, our study was slightly underpowered (0.69) to provide confidence that intake did not increase. Four more subjects would be required to achieve a power of 0.80. On average, girls compensated for approximately 42% and boys for -13% of the energy expended during forty-five minutes of exercise. Similarly, women were found to increase intake following a single bout of high-intensity exercise [22] and moderate to large amounts of repeated exercise [23,24] for up to 19 days [25] but a lack of compensation has been observed in men after two bouts of high-intensity exercise [19], or even after completing three 40 min exercise sessions per day for 7 days [26].

The greater ability of girls to compensate for energy deficits is also suggested by the observation that appetite ratings were strongly correlated with the amount of food consumed by girls, but much weaker in boys (Tables 4 and 5). In girls, subjective appetite scores were positively correlated with food intake at each time point for both SD (r > 0.48) and LD (r > 0.38) sessions. In boys, subjective appetite was only correlated with food intake at fifteen minutes (r = 0.40, p < 0.05) during the LD, but not during any of the SD measurement time points. This may reflect a higher sensitivity to appetite or greater care during completion of VAS questionnaires in girls. In boys, correlation analysis suggests that increased food intake is more strongly related to increased body weight, fat free mass, aerobic fitness (VeT) and levels of disinhibition.

The lack of effect of exercise on food intake was not explained by water intake at the test meal, but provides some indication that the children were more sensitive to physiologic signals of thirst than to hunger after exercise. Unfortunately, thirst was not assessed in this study. Even though all children were asked to drink 250 mL of water immediately after both rest or exercise periods, which was 30 min before the meal was served, water intake at the meal (Table 2) was higher by an average of 60 ml during LDEX but not SDEX. Thus, it can be suggested that 45 min, but not 15 min of exercise was sufficient to lead to a physiologic signal to rehydrate due to loss of body water [27]. The implication of this observation is that non-caloric beverages should be readily available because thirst could drive excess caloric intake if the beverages available are those containing calories.

Intensity of exercise and perhaps thirst may also explain the contrasting results in subjective appetite found in a previous study conducted by our laboratory when compared to this one. In the study reported previously, average appetite and PFC increased following a submaximal fitness test assessing VeT in 9 to 14 year old boys [6]. The fitness test protocol required progression from very low- to high-intensity exercise of approximately 80 - 85% maximum HR over a period of 12 min. However during the current study, children maintained steady state exercise at the VeT, which corresponds to moderate intensity exercise for 15 min. It may be that a higher level of exercise stimulates appetite in the boys, or perhaps they expressed a feeling of thirst rather than hunger when they completed the appetite rating scales. To determine VeT, subjects are fitted with a two-way non re-breathing valve which can dry out the mouth, in addition to water and electrolytes lost due to perspiration. It has been suggested that confusion of thirst with hunger signals may be the cause of excessive energy intake from caloric beverages [28].

This study provides three reasons to suggest SDEX is preferable over LDEX and less frequent activity in a school setting. First, SDEX at the VeT attenuated the increase in average appetite, desire to eat, and hunger that occurred during resting, which suggests that before lunch may be a good time to encourage a SD exercise program. In contrast, LDEX sessions resulted in an increase in average appetite, desire to eat, and hunger in boys and girls. These reported differential effects on subjective appetite did not affect food intake at a meal served 30 min following exercise. However, it is unknown whether the increase in subjective appetite following LDEX would ultimately result in increased food intake if the subsequent meal were served later.

Second, it is possible that three repeated bouts of SDEX at the VeT over the day would elicit a greater net negative energy balance than 1 bout of LDEX, but the long-term significance of this approach to energy balance has not been determined. In boys, one bout of SDEX resulted in a net negative energy balance of -418 kJ during the study measurement period in comparison to -928 kJ following one bout of LDEX. However, repeating SDEX three times daily could result in a greater net negative energy balance of -1254 kJ in boys. Furthermore, the significance of repeated SDEX compared with 1 bout of LDEX is potentially more beneficial in girls compared with boys. In girls, one bout of SDEX resulted in a comparable net negative energy balance to 1 bout of LDEX (-297 kJ vs. -432 kJ) during the study measurement period. However, repeating SDEX three times daily could result in a larger net negative energy balance of -891 kJ.

Third, subjects perceived LDEX to be "harder" than SDEX, as evidenced by reported RPE values during exercise (Table 3), which means they may be less likely to continue a program employing LD exercise. Reported RPE values ranging from 12 to 14 are generally considered a moderate level of exercise and correspond to work perceived as "fairly light to somewhat hard" [16]. Those values correspond closely to mean values from our study (11 - 15). In addition, mean RPE values from the current study are consistent with a previous study that reported a mean RPE of 13.6 during exercise at the VeT in 11 year old children [29].

Last, our study supports the use of VeT as a target intensity for exercise training programs. Exercise at the VeT is practical for implementation as that intensity can easily be assessed without the use of equipment by using the Breath Sound Check (BSC) or the Talk Test. The BSC refers to an intensity at which one can just hear their breathing [30] indicating that they are training at an exercise intensity within 15% of their own VeT. The Talk Test, describes the VeT as when subjects are "just capable of talking" [30] and has a high correlation with the VeT during both treadmill and cycle ergometer exercise in men and women [31]. Both methods are appealing because they do not require equipment and can be applied to different exercise modalities. Furthermore, the VeT corresponds to a moderate level of exercise, which is the intensity recommended to the public by the Canadian government. The Public Health Agency of Canada recommends that each child and adolescent build up physical activity over a period of a few months to achieve at least 60 more min of daily physical activity [32,33]. In addition, increased amounts of moderate activity is associated with reduced body fat and BMI in children [34] which supports use of this intensity in children and adolescents.

## Conclusion

Neither SD nor LD exercise at the VeT increased short-term food intake and SD exercise attenuated increases in appetite. Thus SD exercise programs in schools may be an effective strategy for maintaining healthier body weights in children.

## Abbreviations

ACSM: American College of Sports Medicine; BMI: Body Mass Index; BSC: Breath Sound Check; HR: Heart Rate; LD: Long-duration; LDEX: Long-duration Exercise; LDRT: Long-duration Rest; PFC: Prospective Food Consumption; RPE: Ratings of Perceived Exertion; SD: Short-duration; SDEX: Short-duration Exercise; SDRT: Short-duration Rest; SEM: Standard Error of the Mean; SKF: Skinfold; VAS: Visual Analogue Scales; VeT: Ventilation Threshold; VO_2_: Oxygen Consumption.

## Competing interests

The authors declare that they have no competing interests.

## Authors' contributions

NCB coordinated and executed the study, performed all statistical analyses, and drafted the manuscript. NB conceived of the study, participated in the design and coordination, and helped to draft the manuscript. SGT participated in the study design and provided the facility for fitness testing. PBP participated in the study design and helped draft the manuscript. RCG contributed greatly to the concepts implemented in this study, and has been a pioneer in the field of exercise science. GHA conceived of the study, supervised NCB, and directed drafting of the manuscript. All authors read and approved the final manuscript.
